# Organic Bone Matrix Component Type I and V Collagen Are Not Destructed in Bisphosphonate-Associated Osteonecrosis of the Jaws

**DOI:** 10.3390/medicina58111690

**Published:** 2022-11-21

**Authors:** Yahya Acil, Jan-Tobias Weitkamp, Henning Wieker, Christian Flörke, Jörg Wiltfang, Aydin Gülses

**Affiliations:** Department of Oral and Maxillofacial Surgery, University Medical Center Schleswig-Holstein, Campus Kiel, 24105 Kiel, Germany

**Keywords:** antiresorptive medication, bone, collagen

## Abstract

*Background and objectives*: The investigation of the pathophysiology behind medication-related osteonecrosis (MRONJ) of the jaw mostly focuses on alterations in osteoclast and osteoblast cell activity, but changes in the organic and inorganic bone matrix have rarely been studied. The aim of this study was to investigate whether collagen, the main organic component of extracellular bone matrix, is destructed in osteonecrosis of the jaw secondary to antiresorptive medication. *Material and methods*: Bone samples of patients with MRONJ (*n* = 15, control group *n* = 3) were demineralized, and collagen fragments were separated from intact collagen pellets by ultrafiltration. The quantification of mature collagen cross-links hydroxylysylpyridinoline (HP) and lysylpyridinoline (LP) in pellets and ultrafiltrates was performed by high-performance liquid chromatography (HPLC). The detection of hydroxyproline (Hyp) was carried out using a spectrophotometric assay. In addition, collagen chains were analyzed by sodium dodecylsulfate-polyacrylamide gel (SDS-PAGE). *Results*: The results revealed significantly higher concentrations of HP, LP and Hyp in pellet samples. In addition, there were no significant differences between samples from MRONJ patients and those of the control group. These results were paralleled by SDS- PAGE. *Conclusion*: These findings suggest that MRONJ does not involve the destruction of type I and V collagen molecules, in contrast to previously reported destruction by osteoradionecrosis.

## 1. Introduction

Osteonecrosis of the jaw (ONJ) secondary to antiresorptive medication was firstly described by Marx in oncology patients who received intravenous bisphosphonates in 2003 [1]. Today, in addition to bisphosphonates, it is known that monoclonal antibodies like denosumab and anti-angiogenic agents are also associated with the development of osteonecrosis in the maxilla or mandible [2,3,4]. For this reason, we today refer to ONJ diseases as medication-related osteonecrosis of the jaw (MRONJ) [3]. The aim of antiresorptive medication is to suppress osteoclast activity and therethrough alter bone remodeling to the effect of reducing bone resorption in patients who suffer from osteoporosis, multiple myeloma, cancer bone metastases of solid tumors and some skeletal conditions like Morbus Paget and osteogenesis imperfecta [5]. Hence, patients benefit from the frequency reduction of morbid skeletal events [6,7,8].

However, although the mechanism of action behind bisphosphonates and monoclonal antibodies like denosumab is well studied in vitro and in vivo, the exact etiology behind the development of MRONJ is not yet understood. Bisphosphonates bind to the mineralized bone matrix in the resorption lacunas and are taken up by osteoclasts [5]. The antiresorptive effects are due to suppression of bone resorption [5,9]. Denosumab on the other hand binds specifically to the receptor activator of NF-κB ligand (RANK-L), which results in less bone resorption as well [10]. Nevertheless, osteoclast activation is also the first event initiating bone remodeling, which is believed to be critical for bone viability [11,12]. In context with chronic invasive dental diseases and thin mucosa over bone in the oral cavity, this might predispose to the development of osteonecrosis of the jaw [13,14]. Histopathological findings are empty bone lacunas, bone sclerosis, acute and chronic inflammation with infiltrations of immune cells, bacteria, and poorly vascularized bone [15,16,17]. This can result in great avital areas of jawbone, which then require decortication with the aim to stop further progression.

While many studies focus on cell function and alteration of bone remodeling, changes in the organic and inorganic bone matrix components under MRONJ have been poorly studied. Bone is mainly composed of type I collagen stiffened by crystals of calcium hydroxyapatite. Collagen fibers are responsible for bone flexibility, while the amount of mineralization determines the stiffness. Together they form a pressure- and bending-resistant construct.

It is known that the collagen fiber network is destructed in bone osteoradionecrosis, as our study group previously reported [18]. Similar investigations are not described in the literature for MRONJ.

The aim of this study was to investigate whether type I and V collagen is destructed in osteonecrotic bone samples of patients with MRONJ.

## 2. Materials and Methods

### 2.1. Bone Sample Obtainment

Bisphosphonate-associated osteonecrotic bone samples were obtained from *n* = 15 patients (male *n* = 8; female *n* = 7) undergoing decortication (mandibular *n* = 14; maxillary *n* = 1; mean age in years: 73.8 ± 8.74) in the period of September 2015 until May 2018 at the Department of Oral and Maxillofacial Surgery University Medical Center Schleswig-Holstein, Campus Kiel (Table 1). Patient’s informed consent was obtained of every donor before surgery. All experimental methods were performed in accordance with ethical approval of the local committee of Kiel University (D494/18). All patients fulfilled the clinical MRONJ (stage 2–3) criteria of the American Association of Oral and Maxillofacial Surgeons [3]. Samples were obtained from macroscopically fully destructed and osteonecrotic lesions during surgery. They were only used for further analysis after histopathologic validation of MRONJ postoperatively. Patients were treated with sole Zometa^®^, Aredia^®^, Pamidronat^®^ (all Novartis, Basel, Switzerland) and Ibandronate, or with a cotreatment of two bisphosphonates due to multiple myeloma, prostatic cancer, lymphoma, breast cancer or osteoporosis (Table 1). Mandibular bone samples of *n* = 3 healthy donors served as a control group (mean age in years: 71.67 ± 3.05). Bone samples were obtained during surgery and immediately frozen and stored at −20 °C until further analysis.

### 2.2. Separatin of Broken Collagen Fraction from Intact Collagen Molecules

Firstly, bone samples were dialyzed and demineralized in 0.5 M EDTA solution (pH 7.6) over 3 weeks to remove inorganic matrix compounds. EDTA was then removed by placing bone fragments in 0.5% acetic acid for 2, 6 and 12 h. Acetic acid was renewed three times during this process (2, 6 and 12 h). Finally, samples were lyophilized and stored for further analysis. Broken collagen (chain domains with a molecular weight < 100.000 D) was separated from intact collagen molecules using ultrafiltration.

**Figure 1:**
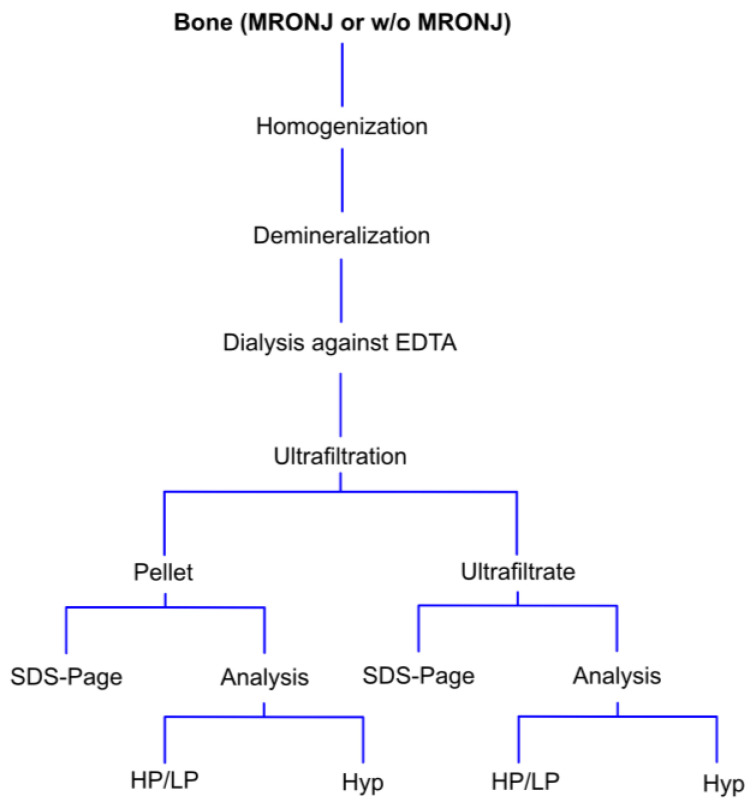
Schematic overview of the analytic process. HP = hydroxylysylpyridinoline, LP = lysylpyridinoline, Hyp = hydroxyproline.

Briefly, lyophilized bone fragments were resuspended in 0.5% acetic acid and transferred to an ultraspin microfilter (Roth, Karlsruhe, Germany) and centrifuged for 30 min at 4000× *g* at room temperature. The ultrafiltrate and the pellet (>100.000 D) were then removed and lyophilized. Both collagen fractions (ultrafiltrates and pellets) were then analyzed according to Figure 1 (see below). By ultracentrifugation, broken and intact collagen were separated following the quantification of mature non-reduceable collagen cross-links hydroxylysylpyridinoline (HP) and lysylpyridinoline (LP) by high-performance liquid chromatography (see below). HP and LP were then used as surrogate parameters to evaluate collagen portions in both phases obtained by ultracentrifugation.

In addition, SDS-PAGE was performed to identify type I and V collagen chains. Type I collagen was investigated because it is the predominant collagen in bone tissue, and type V collagen was additionally investigated as it is characteristic for bone collagen fibrils and co-distributes with type I collagen as the minor component of fibrils.

### 2.3. High-Performance Liquid Chromatographic Analysis of HP and LP

The HP and LP contents of pellet and ultrafiltrate samples with and without MRONJ were hydrolyzed and quantified by high-performance liquid chromatography (HPLC) using external standards as previously described [19,20,21,22]. Briefly, 1 mL of each hydrolysate was mixed with 1 mL acetic acid, 5 mL 10% CF-1-slurry (fibrous cellulose powder, Whatman, Maidstone, UK) and 2 mL n-butanol. The CF-1- slurry was composed of 10% (*w*/*v*) CF-1 in a mobile phased containing n-butan-1-ol, glacial acetic acid and water (4:1.1). A column was prepared by adding the mixture of hydrolysate and CF-1-slurry as described above to an Econo-Column polyprop (40 × 8 mm, Bio-Rad, Munich, Germany). The resin was washed three times with 5 mL of the mobile phase. Subsequently, the pyridinium-containing eluate was eluted from the column with 3 × 2 mL distilled water into a 15 mL plastic tube, and traces of n-butan-1-ol were removed from the surface of the eluate. Thereafter, the lyophilized eluate was redissolved in 1 mL 0.22% (*v*/*v*) n-heptafluorobutyric acid and centrifuged at 750× *g* for 5 min. A total of 200 µL of the sample was analyzed. Finally, HPLC analysis was performed on a Dionex HPLC system (Idstein, Germany). The flow rate was 0.7 mL/min using two continually degassed solvents: solvent A, 0.22% (*v*/*v*) n-heptafluorobutyric acid in water, and solvent B, 0.22% (*v*/*v*) n-heptafluorobutyric acid in 80% (*v*/*v*) acetonitrile. The resin (Inertsil ODS-3 5 mm, 125 mm 9 4.6 mm C18) was equilibrated with 18/82% solvent B to solvent A before application of the sample (200 ll in 0.22% (*v*/*v*) n-heptafluorobutyric acid). The column was washed with 18/82% (*v*/*v*; solvent B/solvent A) for 5 min and developed with the following step gradients: 1. 18–20% solvent B over 20 min; the peaks of HP and LP were eluted at approximately 18 and 20 min; 2. 20–25% solvent B for 4 min; 3. 25–100% solvent B for 1 min plus washing of the column for another 5 min with 100% solvent B; 4. 100–18% over solvent B for 4 min; and 1 min was used for column equilibration thereafter. The next sample was injected after 35 min. Fluorescence was measured with an excitation wavelength of 297 nm and emission wavelength of 397 nm. The concentrations of HP and LP were expressed in nmol/mL.

### 2.4. Quantification of Hydroxyproline

The hydroxyproline in pellets and ultrafiltrates of patients with and without MRONJ was quantified using a custom assay as previously described [18]. Samples were hydrolyzed in 6M HCl, and a volume of 10 µL of each hydrolysate was used for the assay. The assay was carried out using 96-well plates (Corning, New York, NY, USA). Firstly, a standard was prepared by using hydroxyproline ranging from 1 to 5 µg/mL in distilled water. Then, 13 µL per well of samples or standard solution was pipetted. Then, 70 µL in the ratio of 2:1 propan-2-ol to distilled water was added to each well followed by 48 µL chloramine T reagent (Sigma-Aldrich, St. Louis, MO, USA) in 5.6 mg/mL acetate citrate buffer (Roth, Karlsruhe, Germany; pH 6.0). The plate was sealed and incubated at room temperature for 5 min. Finally, 125 µL of Ehrlich’s reagent (24 g dimethylaminobenzaldehyde, 36 mL 60% perchloric acid and 200 mL propan-2-ol) was added. The plate was sealed again and incubated at 70 °C for 10 min. The measurement of absorption was carried out using a flow-through spectrometer at 550 nm.

### 2.5. Detection of Type I and V Collagens Using Dodecyl Sulfate Polyacrylamide Gel Electrophoresis

All samples were lyophilized and dissolved in 5 mL 0.5% acetic acid solution. A total of 250 µL was extracted and lyophilized again. For sodium dodecyl sulfate-polyacrylamide gel electrophoresis (SDS-PAGE) analysis, the lyophilized samples were solved in a sample buffer of 6.25 mL 1 m Tris (pH 6.8), 2.5 g SDS, 10 mL glycerol, 25 mL distilled water, and 3.75 mL 0.25% bromophenol blue and exposed for 3 min to 95 °C. The SDS-PAGE was performed as previously described [19,21]. Three different fetal pig tissues as collagen standards were used: fetal bone, fetal skin and fetal cartilage, respectively, as described in [19]. These samples were prepared with an additional pepsin digestion. In addition to pellets and ultrafiltrates, SDS-PAGE was also performed with the homogenate before centrifugation of samples. Chains of type I collagen: α2 (I), α1 (I), β12 (I), β11 (I) and type V collagen: α2 (V) were identified due to their electrotrophic mobility as described previously (e.g., β11 (I) complexes are at the top of the gel membranes with a molecular weight of approximately 300 kDa, α1 (I) chains 140–160 kDa) [19].

### 2.6. Statistics

All data were tested for normality using the Kolmogorov–Smirnov test. Statistical analysis was performed using Graph Pad prism 5 program (Graph Pad Software Inc., San Diego, CA, USA). One-way ANOVA analysis with Bonferroni’s multiple comparison was used to compare means among the independent experimental groups (collagen markers in the control and MRONJ samples and pellet versus ultrafiltrates). Differences were considered statistically significant if *p* ≤ 0.05. Quantitative data in the text are presented as mean and SD. A post-hoc analysis revealed a power of 0.84.

## 3. Results

### 3.1. Quantification of Collagen Cross-Links HP, LP and Hyp

**Figure 2:**
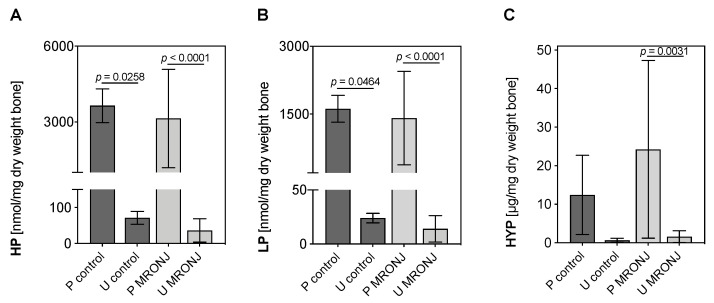
Quantified hydroxylysylpyridinoline (HP) (**A**), lysylpyridinoline (LP) (**B**) and hydroxyproline (HYP) (**C**) concentration in pellets (P) and ultrafiltrates (U) of control and MRONJ samples. Concentrations of HP and LP were detected using HPLC, and Hyp was quantified using a customized standard assay. One-way ANOVA. Data are presented as mean + SD (MRONJ *n* = 15, control *n* = 3).

**Figure 3:**
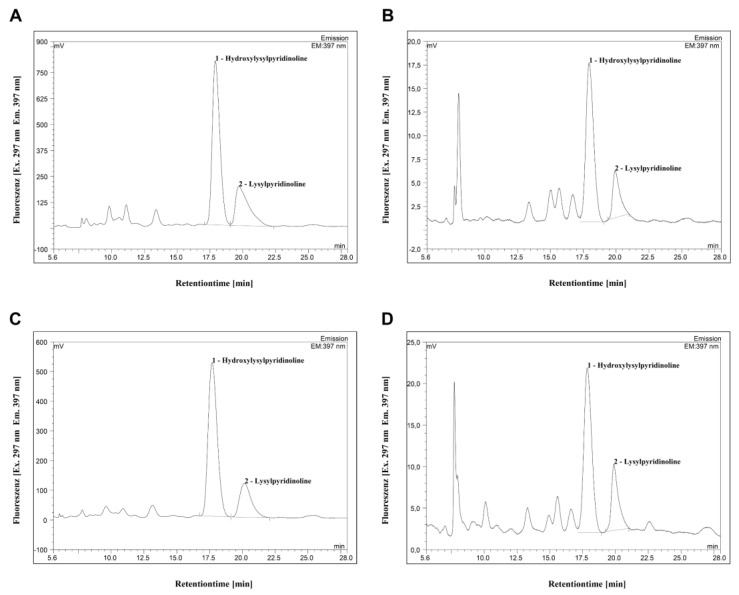
Representative chromatograms of pellets (**A** + **C**) and ultrafiltrates (**B** + **D**) of control (**A** + **B**) and MRONJ (**C** + **D**) samples. Fluorescence was detected at 297 nm (excitation) and at 397 nm (emission). HP peak was measured 17.5 min after injection, followed by the LP peak. Quantification of HP and LP in nmol/dry weight bone of pellets and ultrafiltrates corresponds to the area under the peaks. Quantitative data of healthy and MRONJ were then compared, as shown in Figure 2.

HPLC revealed significantly higher portions of collagen cross-links HP and LP in pellets compared to the ultrafiltrate, indicating intact collagen molecules in bone samples of healthy and MRONJ patients (Figure 2 and Figure 3).

Quantification using HPLC of HP in pellets was significantly higher than those of ultrafiltrates in both control and MRONJ groups (pellet control [3647 ± 668.22 nmol/mL] vs. ultrafiltrate control [71.67 ± 17.79 nmol/mL]: *p* = 0.0116; pellet MRONJ [3139.13 ± 1946.33 nmol/mL] vs. ultrafiltrate MRONJ [36.38 ± 32.43 nmol/mL ]: *p* < 0.0001; Figure 2A), while there was no significant difference detected when comparing pellets and ultrafiltrate groups in direct comparison. A similar pattern was observed for quantified LP in pellets and ultrafiltrates. Collagen cross-link LP was significantly more detected in pellet groups (pellet control [1618 ± 296.64 nmol/mL] vs. ultrafiltrate control [24 ± 4.36 nmol/mL]: *p* = 0.0464; pellet MRONJ [1410.06 ± 1032.44 nmol/mL] vs. ultrafiltrate MRONJ [14.06 ± 12.17 nmol/mL]: *p* < 0.0001; Figure 2 B). In addition, Hyp content in MRONJ groups revealed a similar pattern (pellet MRONJ [24.24 ± 23.04 µg/mg dry weight bone] vs. ultrafiltrate MRONJ [1.59 ± 1.53 nmol/mg dry weight bone]: *p* = 0.0031; Figure 2 C).

In summary, the biochemical analysis of extracellular matrix compounds by HPLC and Hyp assay demonstrated intact type I collagen in samples from both healthy and MRONJ patients. At least > 95% of type I collagen cross-links residues HP, LP and Hyp were detected in pellets of all experimental groups (total amount of HP, LP and Hyp in % minus control samples).

### 3.2. SDS-PAGE of Type I and V Collagen

**Figure 4:**
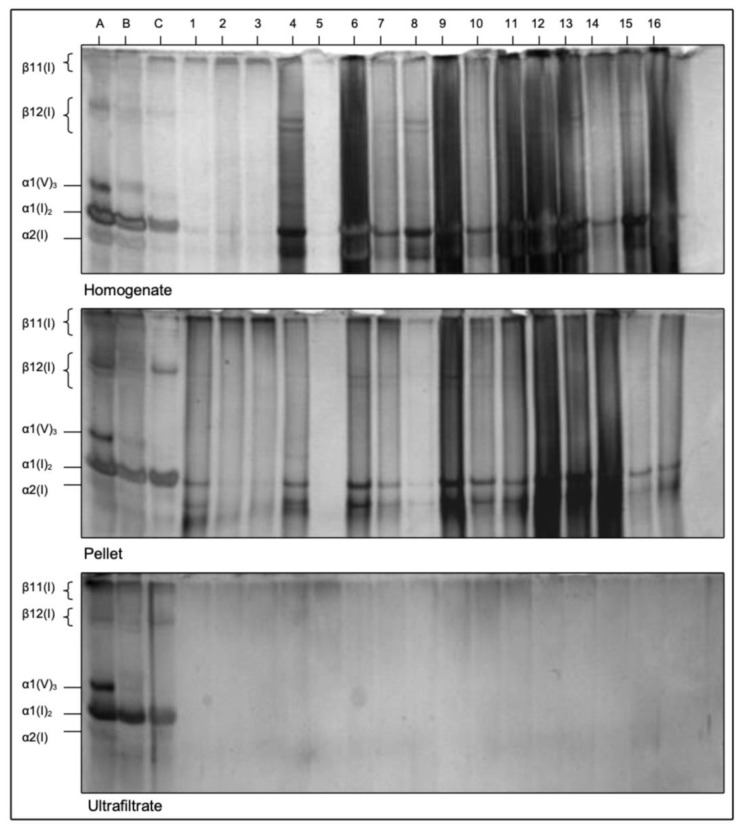
SDS-PAGE of homogenates, pellets and ultrafiltrates of control and MRONJ samples. A-C internal assay controls using fetal pig standard collagens (A bone, B skin, C cartilage); 1–3 control samples, 4–16 MRONJ samples. Gel electrophoresis bands display different collagen chain components of type I and type V collagen. α2 (V) is not shown because it has the same electrotrophic mobility as α1 (V) and about the same molecular weight.

The electrophoretic patterns in SDS-PAGE of α- and β-chains of type I and type V collagen underlined the previously stated results. In the homogenate and pellets, the corresponding collagen bands of controls and MRONJ samples can be identified according to the internal assay control A-C (Figure 4). No collagen residues were detected in the ultrafiltrates. Type I and type V collagen chains (type I collagen: α2 (I), α1 (I), β12 (I), β11 (I), type V collagen: α2 (V)) indicated intact collagen in MRONJ samples.

## 4. Discussion

The aim of this study was to investigate whether type I and V collagen is destructed in patients with MRONJ. Therefore, 15 donors with clinically and histopathologically confirmed osteonecrosis of the jaw secondary to bisphosphonate medication were analyzed and compared to a control group of bone of three healthy patients.

The main finding of this study is that type I and V collagen appears to be significantly more intact than destructed in bisphosphonate-associated osteonecrosis of the jaw. We therefore hypothesize that MRONJ does only marginally affect the organic portion of mandibular and maxillary bone.

Quantification of mature collagen cross-links HP and LP using HPLC showed no significant differences in bone samples of healthy and MRONJ patients. The absence of these covalent intermolecular cross-links in ultrafiltrates indicated intact cross-linked collagen since the molecular weight of collagen molecules is approximately 250–300 kDa and the filter columns used in this study had a cutoff at < 100 kDa [23]. In connection with the detection of intermolecular HP and LP, as previously published by our study group, these results suggest that collagen molecules and intermolecular cross-links are still present in osteonectrotic bone areas [18,19,20,21]. HYP quantification showed the same tendency, although it is not a bone-specific collagen cross-link. The SDS-PAGE results, however, underscored the findings of the above-mentioned investigations. Bands of collagen chains α2 (I), α1 (I), β12 (I), β11 (I) and α2 (V) were observed in homogenate and pellet samples. Ultrafiltrates showed no bands, which is why one can conclude that no collagen chains and therefore no collagen fragments were contained in these samples.

It is well known that the rate and formation of both enzymatic and non-enzymatic collagen cross-linking can change with increasing age, which could reduce the inherent quality of the bone tissue in the elderly skeleton [24]. In order to minimize the age-related effect on collagen concentration, the study sample was based on the data of patients with a mean age of 74.8 ± 8.74 years. The risk of MRONJ could be related to accompanying systemic diseases such as diabetes or medications, such as steroid or immunosuppressive therapy [3]. One might also proclaim that the differences in antiresorptive medication could have an influence on the results of the current study, for example, different half-lives. In order to overcome the medication-related facts, we have decided to include the data of patients that underwent a similar antiresorptive treatment regime (administration of zoledronic acid) without the application of other medications that could affect the bone-turnover and/or increase the risk of MRONJ development.

Our findings also suggest that the pathophysiology of MRONJ may differ from osteoradionecrosis although the present study does not directly compare MRONJ with ORN samples. In a previous study using a similar experimental analytic approach, the direct radiogenic destruction of collagen was reported [18]. However, it is known that in MRONJ patients bone quality is also decreased, although the organic portion seems not to be directly affected [25]. Nevertheless, the bone of patients with MRONJ might be more likely to be returned to health than the bone in osteoradionecrosis, in which the radiation injury relates to the extracellular bone matrix, vascularization and cell populations [26].

In the literature, it has been stated that collagen degradation is regulated by matrix metalloproteinases (MMPs) [27]. Ben-David et al. have indicated that inflammatory processes may trigger osteoblasts to absorb bone by secreting elevated levels of MMPs capable of degrading collagen type I, especially MMP-9 [28]. Despite several studies suggesting that bisphosphonates could increase the amount and enzymatic activity of MMP-9, the role of MMPs in MRONJ could not be confirmed [29]. In the current study, MMPs were not evaluated, however, the results expressed herein might be explained by the suggestions of the abovementioned studies.

A promising treatment strategy to archive bone healing in MRONJ is the use of mesenchymal stem cells (MSCs), due to their immunomodulatory, anti-inflammatory and reparative effects [30]. There are a few pre-clinical studies that support the administration of MSCs. Interestingly MSC-derived exosomes contain a variety of proteins and nucleic acids and hold great potential for promoting cutaneous wound healing, such as in the mucosa of the oral cavity [31]. Therefore, transplantation of MSCs to the osteonecrotic defect site is a promising strategy to return bone to health using the present organic matrix.

With increasing doses of bisphosphonates, moreover, the cell populations from which bone regeneration can originate are increasingly inhibited. For example, higher doses of zoledronate inhibit type I collagen expression in periodontal ligament stem cells [32]. This also suggests a reuse of collagens in the bone healing of MRONJ defects.

It is well known that serum and urinary CTX (C-terminal crosslinked telopeptide of type I collagen) values are suggested to be efficient markers of bone resorption by increased osteoclastic resorptive activity in many metabolic bone diseases. Therefore, it has been hypothesized that CTX tests could have a predictive value in determining the risk of osteonecrosis in patients taking bisphosphonates. However, later studies suggest that CTX testing might not be a sensitive indicator in predicting risk of MRONJ [33,34,35,36]. The results of the current study also confirmed that the assessment of CTX values could not be a predictable tool in determining the risk of MRONJ, since the organic portion of the jaws remains unaffected.

The limitation of the present study is that the macro architecture of the bone collagen network in MRONJ was not investigated and therefore it allows for no statement about the functional properties of the extracellular matrix. In addition, changes in cellular activity, e.g., MMP secretion, were not evaluated. Here, further research is necessary to fully understand the complex changes in bone matrix alterations in MRONJ lesions.

## 5. Conclusions

Bisphosphonate-associated osteonecrosis of the jaw does not directly involve the destruction of organic extracellular matrix compound collagen in the jawbone. Nevertheless, changes in bone micro architecture and infection result in reduced bone quality. In the case of bone healing, however, bone matrix components could be reused by remodeling processes.

## Figures and Tables

**Table 1:** Overview of donors’ age, sex, diagnose and antiresorptive medication.

ID	Birth Year/Sex		Diagnosis	Donor Site	Bisphosphonate
1	1940	♀	Multiple Myeloma	Mandible	Zometa^®^ + Aredia^®^
2	1966	♂	Prostate cancer	Mandible	Zometa^®^
3	1943	♀	Breast cancer	Maxilla	Zometa^®^ + Aredia^®^
4	1949	♂	Multiple Myeloma	Mandible	Zometa^®^ + Pamidronat
5	1949	♂	Lymphoma	Mandible	Zometa^®^ + Ibandronat
6	1945	♀	Breast cancer	Mandible	Zometa^®^
7	1949	♂	Multiple Myeloma	Mandible	Zometa^®^ + Aredia^®^
8	1959	♀	Multiple Myeloma	Mandible	Zometa^®^
9	1934	♂	Multiple Myeloma	Mandible	Zometa^®^ + Ibandronat
10	1938	♀	Breast cancer	Mandible	Aredia^®^
11	1923	♀	Osteoporosis	Mandible	Zometa^®^ + Pamidronat
12	1949	♀	Multiple Myeloma	Mandible	Zometa^®^ + Ibandronat
13	1933	♂	Multiple Myeloma	Mandible	Zometa^®^ + Pamidronat
14	1939	♂	Multiple Myeloma	Mandible	Zometa^®^ + Pamidronat
15	1937	♀	Osteoporosis	Mandible	Zometa^®^ + Pamidronat
Control Group (*n* = 3)					
	1947	♂		Mandible	No antiresorptive medication
	1945	♀		Mandible	No antiresorptive medication
	1941	♀		Mandible	No antiresorptive medication

## Data Availability

The datasets used and/or analysed during the current study available from the corresponding author (jan-tobias.weitkamp@uksh.de) on reasonable request.
